# The Surgery of the Lung

**Published:** 1886-01

**Authors:** 


### The Surgery of the Lung.

Dr. H. Truc, of Paris, has recently published an exhaustive article on this subject. He concludes that pneumotomy seldom gives favorable results in tubercular affections, but claims that it may be advantageously employed—

1. In abscess.
2. In circumscribed gangrene.
3. In severe localized putrid bronchitis.
4. In large hydatid cyst of the lung.
5. For the removal of foreign bodies from the lung.
6. In some cases of pulmonary cancer.

The author claims that exploratory puncture are generally harmless. In operating, he prefers the thermo-cautery.—*Journal de Med. de Paris.*

